# A Comparison of Administrative and Physiologic Predictive Models in Determining Risk Adjusted Mortality Rates in Critically Ill Patients

**DOI:** 10.1371/journal.pone.0032286

**Published:** 2012-02-24

**Authors:** Kyle B. Enfield, Katherine Schafer, Mike Zlupko, Vitaly Herasevich, Wendy M. Novicoff, Ognjen Gajic, Tracey R. Hoke, Jonathon D. Truwit

**Affiliations:** 1 Department of Medicine, University of Virginia, Charlottesville, Virginia, United States of America; 2 Hospital Epidemiology, University of Virginia, Charlottesville, Virginia, United States of America; 3 Department of Medicine, Cleveland Clinic, Cleveland, Oklahoma, United States of America; 4 Department of Medicine, Mayo Clinic, Rochester, Minnesota, United States of America; 5 Department of Public Health Sciences, University of Virginia, Charlottesville, Virginia, United States of America; 6 Department of Pediatrics, University of Virginia Children's Hospital, Charlottesville, Virginia, United States of America; University Medical Center Rotterdam, Netherlands

## Abstract

**Background:**

Hospitals are increasingly compared based on clinical outcomes adjusted for severity of illness. Multiple methods exist to adjust for differences between patients. The challenge for consumers of this information, both the public and healthcare providers, is interpreting differences in risk adjustment models particularly when models differ in their use of administrative and physiologic data. We set to examine how administrative and physiologic models compare to each when applied to critically ill patients.

**Methods:**

We prospectively abstracted variables for a physiologic and administrative model of mortality from two intensive care units in the United States. Predicted mortality was compared through the Pearsons Product coefficient and Bland-Altman analysis. A subgroup of patients admitted directly from the emergency department was analyzed to remove potential confounding changes in condition prior to ICU admission.

**Results:**

We included 556 patients from two academic medical centers in this analysis. The administrative model and physiologic models predicted mortalities for the combined cohort were 15.3% (95% CI 13.7%, 16.8%) and 24.6% (95% CI 22.7%, 26.5%) (t-test p-value<0.001). The r^2^ for these models was 0.297. The Bland-Atlman plot suggests that at low predicted mortality there was good agreement; however, as mortality increased the models diverged. Similar results were found when analyzing a subgroup of patients admitted directly from the emergency department. When comparing the two hospitals, there was a statistical difference when using the administrative model but not the physiologic model. Unexplained mortality, defined as those patients who died who had a predicted mortality less than 10%, was a rare event by either model.

**Conclusions:**

In conclusion, while it has been shown that administrative models provide estimates of mortality that are similar to physiologic models in non-critically ill patients with pneumonia, our results suggest this finding can not be applied globally to patients admitted to intensive care units. As patients and providers increasingly use publicly reported information in making health care decisions and referrals, it is critical that the provided information be understood. Our results suggest that severity of illness may influence the mortality index in administrative models. We suggest that when interpreting “report cards” or metrics, health care providers determine how the risk adjustment was made and compares to other risk adjustment models.

## Introduction

Risk adjusted mortality rate is one publicly reported metric used to describe hospital quality. Public reporting of hospital mortality began in 1986 with the release of inpatient mortality data for Medicare patients by the Health Care Financing Agency. Consumer groups applauded this effort, but the release of raw mortality rates without risk adjustment was quickly shown to be misleading [1]. In the years that followed, process and outcomes measures have been increasingly used to describe the quality of care received by patients in United States Hospitals [2].

Outcome measures focus on patient events, unlike process measures which evaluate on task performance such as “door to balloon time” in acute coronary syndrome or timing of antibiotic administration for sepsis. Outcome measures include catheter-associated central line infections, hospital readmission, and mortality, among others. Ultimately, these comparisons are designed to result in systematic improvements in healthcare outcomes and delivery, sometimes through financial penalties [3]. Providers are reasonably skeptical of these comparisons due to inherent differences in populations, which may impact observed healthcare outcomes [2].

Differences in unadjusted mortality at any given hospital are dependent on multiple internal and external factors. Adjusting for severity of illness attempts to compensate for these differences [1]–[8]. Risk adjusted mortality is often reported as the mortality index (number of observed deaths divided by expected deaths) for a patient population [9], [10]. The inherent problem with using adjusted mortality is that it requires readily identifiable factors that can be reproducibly and accurately measured [10]. Therefore, while multiple well-designed mortality prediction models exist for acute care and critical care patients, it is possible that two well developed models will produce disparate results. The challenge for consumers of this information, both the public and healthcare providers, is interpreting differences in risk adjustment models [3].

Understanding how models might differ is important when comparing published results. This has recently become evident in the academic literature. Bratzler et al recently reported that administrative claims-based data (an administrative model) for patients admitted with community acquired pneumonia closely estimates mortality risk as predicted using variables extracted from the medical record (a physiologic model) [11]. Similarly, the University Health Consortium recently hosted a webinar (December 5, 2011) on how an administrative based method for identifying central line infections compared to that National Health and Safety Network physiology based method for identifying central line infections.

We sought to determine if, an administrative predictive model would estimate mortality risk similarly to a physiologic predictive model in the population confined to the critically ill. Unlike the study by Bratzler et al [11], we would be assessing a group with multiple diseases and with a high severity of illness. We hypothesized that the administrative and physiologic models would not produced similar risk-adjusted mortality risk estimates in critically ill patients.

## Methods

### Ethics

The University of Virginia (Charlottesville, VA) and Mayo Clinic (Rochester, MN) Institutional Review Boards (IRB) approved the protocol prior to reviewing charts. The IRB at the University of Virginia (UVA) gave permission for waiver of consent given the minimal risk to patients. The IRB at the Mayo Clinic required consent of the patient prior to including their information in the research database. Only those patients who gave permission to use their medical records for research were included at Mayo Clinic database. This project was started at UVA and expanded to the Mayo Clinc. Both medical intensive care units (ICUs) are primarily receive patients from the emergency department, the acute care floors, and by intra-hospital transfer, giving them similar characteristics. In addition these two hospitals have collaborated in the past and both provide data directly to the UHC for quality improvement purposes.

### Model Choice and Calibration

#### Administrative model

For this study the UHC model was chosen as our administrative model. We chose the UHC model because it is internally calibrated and internal validity testing shows it predicts 84% of the odds of death in critical illness (personal communication from Mark Keroak, UHC). Approximately 90% of non-profit academic hospitals in the United States participate in the UHC, which constructed its model to calculate predicted mortality based on patient characteristics collected from claims-based data(University HealthSystem Consortium, Oak Brook, IL, 2009). This administrative model primarily uses comorbidities to predict patient mortality as well as determining severity of illness as assigned by the Diagnostic Related Group (DRG) [5], [12]. The model uses administrative variables coded from clinical documentation entered upon admission and throughout hospitalization [5], [12], [13].

#### Physiology Model

We chose the APACHE IV based on its well known characteristics and availability of free online tools to calculate. The APACHE-IV model uses extremes in physiologic variables during the first 24 hours of admission to an ICU as well as disease specific variables to predict mortality as well as length of stay. The characteristics of the APACHE-IV model are published in the peer-reviewed literature [4], [5], [12].

The models were not calibrated to our data but the intercepts from the original models were used. Calibration of the variables to local mortality at each site would improve the relationship between those variables in our sample; however, by doing so we would not be describing the models as they are applied in “real life” and this would decrease the utility of this study. This approach was chosen to represent as close as possible performance in “real life”.

### Sample

The University of Virginia Hospital is a 534-bed academic medical center in Charlottesville, Virginia. Annually, over 33,000 patients receive inpatient or observation care at the University of Virginia Hospital. The medical intensive care unit (MICU) is a 16 bed closed ICU. The Mayo Clinic is a tertiary care, academic medical center with 1900 beds and 135,000 hospital admissions per year. The combined capacity of the ICUs is 204 beds and 14,800 admissions per year.

At the University of Virginia, we evaluated 200 consecutive MICU patients who were enrolled prospectively over a three-month period (May through July 2007). Two of the investigators (KS, MZ) abstracted the variables from chart review and then calculated APACHE IV scores. A random sample of 1/3^rd^ of the data entries was verified by a third investigator for quality assurance (KE). These variables included the physiologic parameters necessary to calculate APACHE IV scores. UVA obtained UHC mortality predictions directly from UHC using the patients' account numbers, which are unique to each patient and visit.

Beginning on March 21, 2005, the medical records of all new patients coming to the Mayo Clinic campus in Rochester, MN, were stored in an electronic form [14]. The Mayo Clinic performed UHC and APACHE IV score calculations on the random sample of 400 patients admitted to the MICU in 2007. This database includes the same variables that the University of Virginia collected, providing the APACHE IV scores for these patients as well as their UHC mortality predictions. The Mayo Clinic transferred the data in de-identified format to the University of Virginia where all statistical analyses were performed.

### Definitions

Patients were classified as being admitted to the ICU from one of three locales: acute care unit (ward), emergency room, or from an outside hospital. Patients with multiple ICU admissions within the same hospital stay were included, but we analyzed only the primary admission to the ICU. To minimize confounding that might occur in patients transferred to the ICU from the acute care ward or referring facility, we performed a subgroup analysis of patients admitted directly from the emergency department. We coded mortality based on hospital mortality, as it is the ultimate outcome of interest for clinicians. For the purpose of this study we described unexpected mortality within each model separately and defined unexpected deaths as those patients with a predicted mortality less than or equal to 10% who died.

For the purposes of our study, we defined the first twenty-four hours as beginning when the patient physically entered the MICU. This definition thereby occasionally excluded patient variables accrued during the hospitalization, but outside of the physical confines of the MICU. We chose this definition in order to best assess the quality of care in this particular unit and by its staff. It was also felt that the clinical variables, particularly vital signs, were more reliable during this time period because of uniform data collection once the patient was in the ICU. This design element could underestimate the true APACHE IV value because the model uses the worst value in the 24-hour period, and vital signs in the hours preceding ICU admission could have been more deranged.

### Analysis

We performed all statistics with SAS v 9.2 for Windows and SPSS v 20.0 for Macintosh (Chicago, IL). Each model's receiver-operator characteristic was determined and calibration described with the Hosmer-Lemeshow test. The distribution of the predicted mortality was analyzed for normality and non-normally distributed median predicted mortalities were compared by Wilcoxon-rank-sum test. We compared the correlation of the predictive mortality methodologies using Pearson's product moment correlation coefficient. We also generated Bland-Altman^15^ plots by creating two variables in SPSS, BADIFF (UHC mortality- APACHE mortality) and BAMEAN ((UHC mortality+APACHE mortality)/2).

The Mortality index for each hospital was calculated for both the clinical model (APACHE-MI) and administrative model (UHC-MI). Differences between the two hospitals were described by comparison of the mortality index means, as this is how mortality index is typically published, and by comparisons of medians as the data was not normally distributed. Post-hoc we analyzed the difference in variance of predicted APACHE mortality by quartile of UHC predicted mortality. We also analyzed differences in Mortality Index by hospital to further understand how differences in severity of illness may influence these results.

## Results

We included 556 patients from the two academic medical centers in this analysis. The mean age (in years) of the admitted patients was 62. Eighty-nine percent of the patients were white and 58% were male. The average APACHE IV score was 68.58. The observed mortality for the cohort was 18.1%. Using the administrative model there were 20 patients (3.6% of all patients, 19.8% of deaths) with unexpected deaths based on our definition. Using the physiologic model, there were 7 patients (1.3% of all patients and 6.9% of deaths) with unexpected deaths. Table 1 describes the patient population along with their APACHE IV diagnoses.

**Table 1:** Patient Population Characteristics and Diagnosis Frequencies.

	Hospital A	Hospital B	Pooled	p-value
**N = **	164	392	556	
**Mean Age yrs (range)**	57.3(18–94)	63.8(18–104)	61.77(18–104)	**0.798**
**Gender (%male)**	60	52.3	57.8	**<0.001**
**Ethnicity (%white)**	81.9	86.4	84.9	**0.175**
**Mean APACHE IV Score (range)**	66.2(23–132)	69.7(12–161)	68.58(12–161)	**0.153**
**Emergency Department Admissions**	73(46%)	196(50%)	269(48%)	**0.238**
**UHC Predicted Mortality**	0.194(SD 0.208)	0.135(SD 0.172)	0.153(SD 0.185)	**0.056**
**APACHE Predicted Mortality**	0.298(SD 0.251)	0.224(SD 0.211)	0.246(SD 0.225)	**0.001**

APACHE Diagnosis				
	N (%)	N (%)	N (%)	
**Respiratory**				
**- ARDS**	1 (0.6)	5 (1.2)	6 (1.1)	**0.488**
- Respiratory Arrest	3 (1.8)	3 (0.8)	6 (1.1)	**0.268**
- Respiratory- other	43 (26.2)	73 (18.6)	116 (20.9)	**0.044**
- Pneumonia	18 (11.0)	45 (11.5)	63 (11.3)	**0.864**
**Cardiac**				
- Cardiac Arrest	6 (3.7)	4 (1.0)	10 (1.8)	**0.033**
- CARDIAC Other	4 (2.4)	18 (4.6)	22 (4.0)	**0.235**
**GI**				
- GI Bleed	25 (15.2)	47 (12.0)	72 (12.9)	**0.297**
- GI Other	1 (.6)	19 (4.8)	20 (3.6)	**0.014**
**Metabolic**				
- DKA	2 (1.2)	8 (2.0)	10 (1.8)	**0.506**
- ENDO Other	2 (1.2)	10 (2.5)	20 (3.6)	**0.324**
**Renal**				
- Renal Failure - Acute	9 (5.5)	14 (3.5)	23 (4.1)	**0.301**
**Neuro**				
- NEURO Other	3 (1.8)	23 (5.9)	26 (4.7)	**0.397**
- COMA	13 (8.9)	8 (2.0)	21 (3.8)	**0.001**
**Systemic**				
- Overdose	5 (3.0)	33 (8.4)	38 (6.8)	**0.022**
- Sepsis	24 (14.6)	43 (11.0)	67 (12.1)	**0.226**
**Other**	**5** (3.0)	**32** (8.1)	37 (6.7)	**0.028**

**Figure 1:**
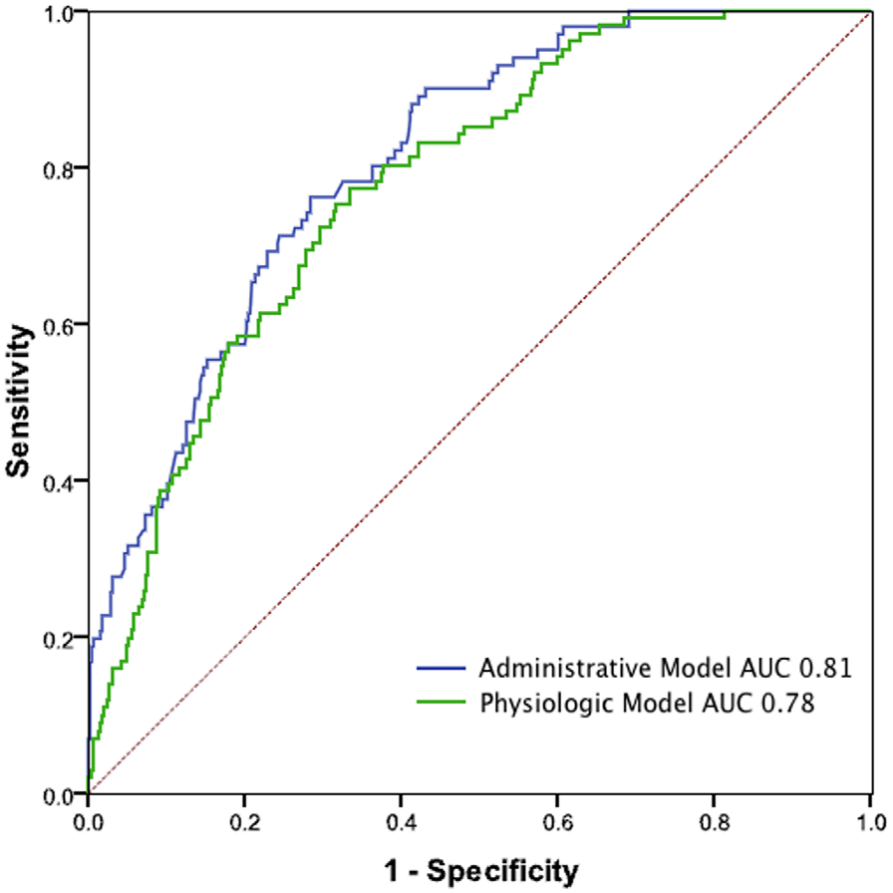
ROC Curve for UHC and APACHE-IV models to discriminate survivors from non-survivors.

The administrative model and physiology model, shown in Figure 1, had an AUC in our cohort of 0.81 (95%CI 0.77, 0.86) and 0.78 (95% CI 0.73, 0.82) respectively. The Hosmer-Lemeshow test of calibration was statistically significant for both the administrative model (p-vale 0.044) and physiologic model (p-value 0.005).

**Figure 2:**
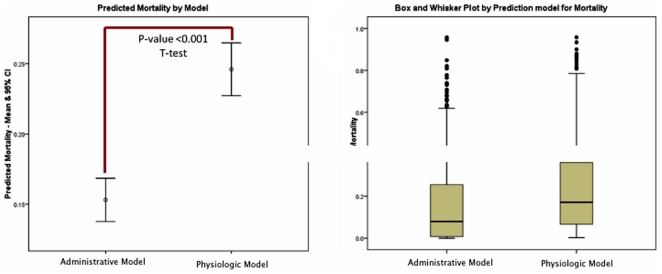
Predicted Mortality by prediction model. Panel A: Mean and 95% CI with T-Test result. Panel B: Box and Whisker Plot for each model.

The administrative and physiologic models' predicted mortalities were not normally distributed. The median predicted mortality for administrative model and physiologic model for the combined cohort were 7.9% (ICR 24.7%) and 17.0% (ICR 29.4%), which were statistically different (p-value<0.001) (Figure 2). The mean ratios of observed to expected mortality (mortality index) for the combined cohort by administrative model and physiologic models were 1.73 and 0.71, respectively.

**Figure 3:**
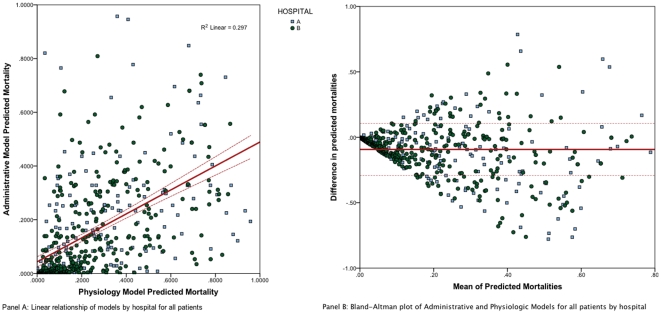
Relationship between UHC and APACHE Models. Panel A. Linear relationship for UHC model (y-axis) and APACHE-IV (x-axis) for subgroup admitted directly from emergency department. Panel B. Bland-Altman Plot of Predicted Mortality for those patients admitted directly to the ICU from the Emergency Department: The x-axis represents the mean of the two values and the y-axis represents the difference.

The two models showed weak correlation (Figure 3 panel A), with a Pearson product-moment correlation coefficient of r = 0.545 (p-value<0.0001). There is a linear relationship between the two values, as shown in Figure 3 panel B, with an r^2^ of 0.297. Although correlation coefficients demonstrate whether or not two measures are related, they do not reflect the presence or absence of agreement [15]. The Bland-Altman plot (figure 3) suggest that these two models are not only poorly correlated (as shown by the Pearson product-moment correlation coefficient) but they also have poor agreement (Figure 3). As predicted mortality increases, separation between physiologic and administrative models widens. However, as the Bland-Altman plot shows, there is wide disagreement between the methods with administrative model being both higher and lower than the physiologic model prediction, and showing greater variation at higher mean predicted mortality. Approximately 6% of the values lie outside of two standard deviations from the mean of the observations.

We also separately analyzed those patients admitted directly to the ICU from the emergency department. In this smaller combined cohort of 269 patients, the administrative model predicted mortality was 12.7% (95% CI 10.5%, 15.0%) and the physiologic model predicted mortality was 20.8% (95%CI 18.4%, 23.2%). The observed mortality in this subgroup was 11.5% (administrative model mortality index .91, physiologic model mortality index 0.61).

**Figure 4:**
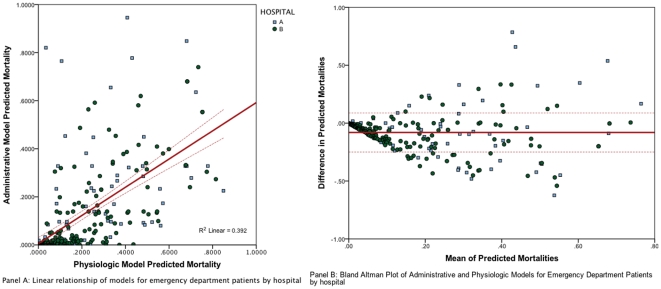
Relationship between UHC and APACHE Models for patients admitted through the emergency department. Panel A. Linear relationship for UHC model (y-axis) and APACHE-IV (x-axis) for subgroup admitted directly from emergency department. Panel B. Bland-Altman Plot of Predicted Mortality for those patients admitted directly to the ICU from the Emergency Department: The x-axis represents the mean of the two values and the y-axis represents the difference.

The correlation between the two samples was statistically significant (p-value<0.0001) with a Pearson Correlation of r = 0.626 and an r^2^ of 0.392, as shown in Figure 4 Panel A. This result suggests better, but still weak, correlation between the two models. Again, the prediction models for this smaller subset show poor agreement as described by the Bland Altman plot displayed in Figure 4 Panel B. In this subset around 8% of the values lie outside of 2 standard deviations from the mean but as in the full sample, there is a significant difference between the two models, which increases as the mean predicted mortality increases.

To better understand the divergence in the Bland-Altman plots, the variance of the physiologic model was plotted in quartiles of administrative model predictive mortality. ANOVA was performed to determine if the variance was the same at each quartile, with the null hypothesis of equal variances. The variance in physiologic model predicted mortality at each quartile of administrative model predicted mortality was statistically significantly, with increasing variance as predicted mortality rose in the administrative model (Table 2).

**Table 2:** APACHE IV Predicted Mortality Mean, Standard Deviation, and Variance by Qaurtile of UHC Predicted Mortality ANOVA (chi-aquare 254.5 p<0.0001) rejected the null hypothesis that the variances where equal.

UHC Quartile	APACHE IV Mean	95%Ci	Standard Deviation	Variance
0–.007	0.06	0.1568	0.08	0.006
.008–.07	0.17	0.3136	0.16	0.026
.08–.25	0.31	0.392	0.2	0.043
.26–.95	0.42	0.4508	0.23	0.055

Comparing hospital 1 (n = 164) and hospital 2 (n = 392), (table 3) there was a statistically significant difference in unadjusted mortality (27.2 and 14.2% respectively, p-value<0.001) with no statistical difference in APACHE IV score (65.9 and 69.7 respectively, p-value 0.11). This difference remained statistically significant (p-value 0.047) when mortality was adjusted using the administrative model. The mortality index for hospital 1 was 2.39 (95% CI 1.11, 5.66) and for hospital 2 1.03 (95% CI 0.53, 1.54). However, when the physiologic model was used there was no difference in the two hospitals mortality index, with hospital 1 having an adjust mortality of 1.03 (95% CI 0.57, 1.49) and hospital 2 having an adjusted mortality index of 0.56 (95% CI 0.39, 0.75)(p-value 0.66). The median mortality index for both models was zero for both hospitals as more than fifty percent of patients survive, so the observed/expected value for most patients is zero. However, the interquartile range, was different for the hospitals and there was a statistical difference for both models (administrative p-value 0.003, clinical p-value 0.0006).

**Table 3:** Hospital Comparison for UCHMI and APACHEMI for full sample and at each quartile of UHC predicted mortality.

	Hospital 1	Hospital 2	p-value
**n**	**164**	**392**	
Raw Mortality	27.20%	14.20%	<0.0001
APACHE Score	65.9	69.7	0.1057

		Mean (95% CI)	Median (IQR)	Mean (95% CI)	Median (IQR)	Parametric	Non-parametric
	Administrative Model Mortality Index	3.39 (1.11, 5.66)	0.00 (1.25)	1.03 (0.53, 1.54)	0.00 (1.18)	0.047	0.0003
	Clinical ModelMortality Index	1.03 (0.57, 1.49)	0.00 (1.18)	0.56 (0.39, 0.75)	0.00 (0.00)	0.066	0.0006
**By UHC Predicted Mortality Quartile**							
Q1	n	21		111		132	
	Mean APACHE IV Score	49.14		48.37			
	Administrative Model Mortality Index	0	0.00 (0)	0	0.00 (0)	NA	NA
	Clinical ModelMortality Index	0	0.00 (0)	0	0.00 (0)	NA	NA
Q2	n	37		101		138	
	Mean APACHE IV Score	54.9		65.8			
	Administrative Model Mortality Index	10.66 (0.71, 20.59)	0.00 (0)	1.98 (0.11,3.77)	0.00 (0)	0.08	0.02
	Clinical ModelMortality Index	0.75 (0.01, 1.51)	0.00 (0)	0.35 (0, 0.72)	0.00 (0)	0.28	0.03
Q3	n	53		85		138	
	Mean APACHE IV Score	72.5		77.8			
	Administrative Model Mortality Index	2.13 (1.11, 3.15)	0.00 (4.29)	1.45 (0.73, 2.17)	0.00 (0)	0.26	0.15
	Clinical ModelMortality Index	0.93 (0.35, 1.51)	0.00 (1.18)	0.67 (0.29, 1.07)	0.00 (0)	0.44	0.16
Q4	n	53		95		148	
	Mean APACHE IV Score	73.6		91.5			
	Administrative Model Mortality Index	0.92 (0.59, 1.26)	0.00 (1.52)	0.92 (0.66, 1.18)	0.00 (2.09)	0.98	0.84
	Clinical ModelMortality Index	1.72 (0.52, 2.92)	0.00 (1.66)	1.37 (0.88, 1.86)	0.00 (1.82)	0.52	0.74

**Figure 5:**
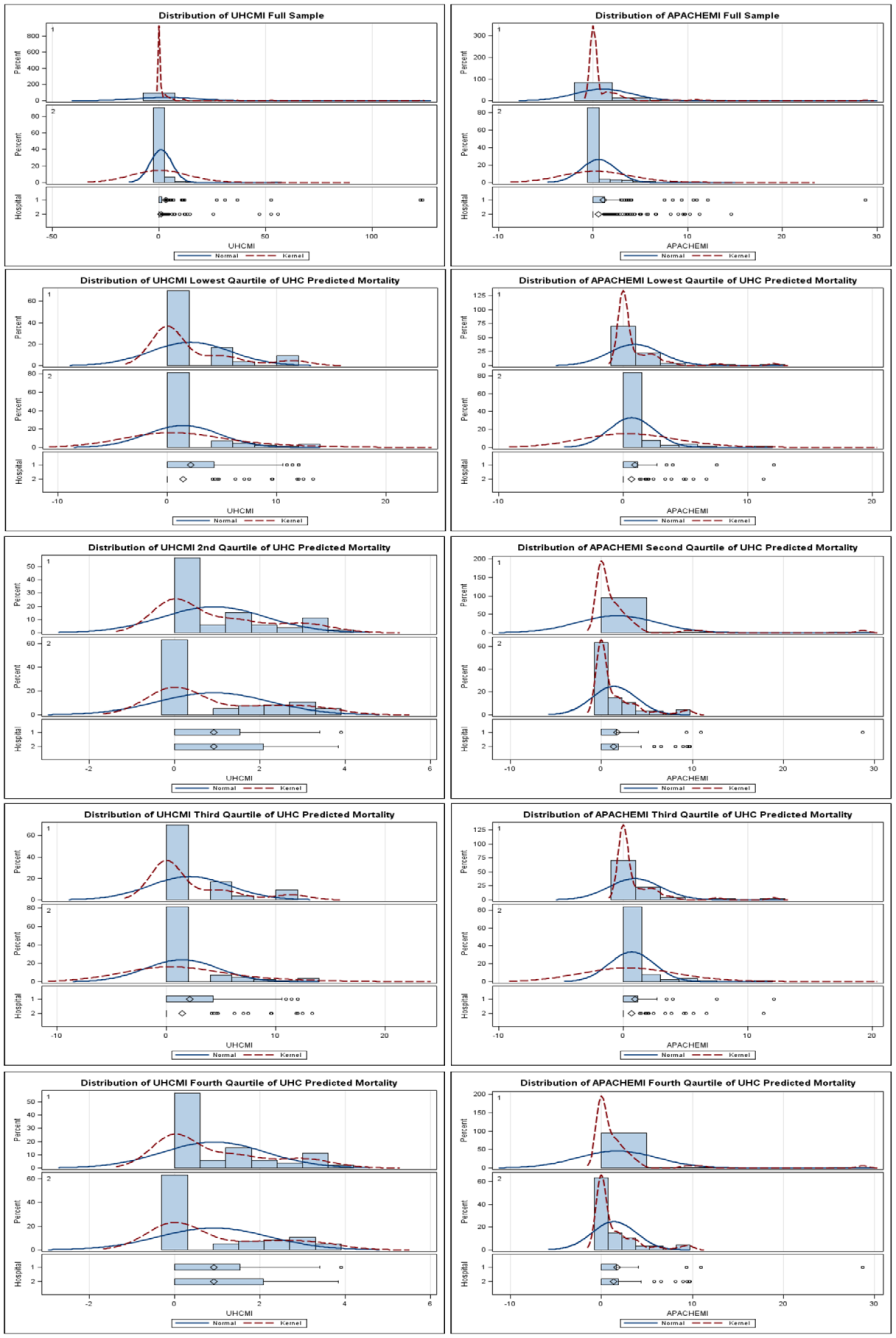
Distribution of the UHCMI and APACHEMI by hospital, compared for the total poulation and at each qaurtile of UHC predicted mortality.

Further dissection of the two models shows that as average predicted mortality increases, as defined by our administrative model, the hospitals outcomes become statistically the same, both in comparison of means and medians (Table 3). As predicted mortality increases (Figure 5), the distributions of mortality index become similar between the two hospitals. We note that at the lowest quartile of predicted mortality, no patients died. At the next two quartiles, outliers in both hospitals have significant impacts on the mean mortality index, and at the highest quartile the mean administrative model mortality index is 0.92 at both hospitals.

## Discussion

In our analysis, we demonstrate in a population of medical ICU patients across two academic health centers, that the reported mortality index for this sample ranged from 0.71 (physiologic model) to 1.73 (administrative model) depending on the model chosen for reporting. Furthermore, when comparing the two institutions the models differ in their conclusion. The administrative model suggests there is a statistical difference in outcomes between Hospital 1 and 2. The physiologic model suggests there is no statistical difference between the two hospitals. This information could lead providers to inaccurate conclusions with regard to the quality of their practice or institution. Likewise, patients presented with this information may be influenced in different ways by these conclusions.

Importantly our results also suggest that in critically ill patients, the administrative model is influenced by the severity of illness in a way that goes against the goal of risk adjustment. The risk-adjusted mortality for both hospitals is well above one when the predicted mortality for the patients is very low. Conversely, at higher levels of predicted mortality, the mortality index at both hospitals approached 1. So if a hospital's patients are oversampled for high predicted mortality the hospital mortality index may be very close to one, however, if their population is oversampled for patients deemed to have a low risk of death, hospital mortality index could easily exceed one. This is counter to the goal of the mortality index, which is to allow for hospital comparisons.

To our knowledge this is the first study to describe the practical application of two widely used mortality prediction models in critically ill patients that differ in methodology. Previous literature in critically ill patients has focused on different physiologic models [6], [16]. Our study does not support the conclusion that one model is superior or should be chosen over another. It builds on the work of Kuzniewicz et al by demonstrating important facts about standardized mortality rates [16]. First, the methodology used to develop a mortality prediction model can influence how those models compare. Second, two models can have similar AUC and still perform remarkably differently. As described by Bland and Altman, the correlation of two methods designed to measure the same parameter or property does not automatically imply that there is good agreement [15].

There are important differences in our study from what was published by Bratzler et al [12]. First, their study did not focus on critically ill patients. Second, they focused on patients admitted with a diagnosis of community acquired pneumonia. These population differences could explain the differences in our results. Importantly, the patients in our study with a low probability of death as predicted by our administrative model (UHC) had similar predicted mortality by the physiologic model (APACHE). This result is reflected in our finding that variance in physiologic model predicted mortality increased as administrative model predicted mortality increased.

The discrepancies between the two models may reflect limitations associated with this study. First, while this study is multicenter, our institutions share common features in that they are both academic medical centers providing tertiary and quaternary care to a wide referral base. Therefore, our results may not be generalizable to all ICUs. Second, while relatively large, our sample size is small compared to the validation cohorts used by both UHC and APACHE. It is also important to note that our study may still under represent the administrative model because it relies on coded variables, which may reflect similar limitations in documentation between institutions. Unlike hospital 2, hospital 1 did not utilize and electronic database of critically ill patients. The choice of 200 consecutive patients may reflect another potential source of bias; however, we have previously shown that there is little seasonal influence at our institution on outcome [17].

It should be noted that the appeal of administrative modeling is the relative ease of collecting and analyzing the variables in the model. However, there are important biases introduced when using physician-coded diagnosis for risk adjustment, specifically the accuracy of those diagnoses. Additionally, the coding may not adequately characterize critically ill patients. For example, two patients admitted with pneumonia may be very different in their degree of physiologic derangement and this discrepancy may not be fully captured in administrative modeling. Our study may also be impacted by discharging patients from a hospital who ultimately die at a long term acute care hospital (LTACH) which hospital 2 had at the time of the study and hospital 1 did not at the time of the study. Alternatively, in the absence of a robust electronic tool, physiologic based modeling is labor intensive and may be difficult to apply across multiple hospitals without first addressing differences in infrastructure.

In our studies, both models were outstanding at this discrimination, as described by the ROC curve in Figure 1. Interestingly, both models had significant Hosmer-Lemeshow tests, suggesting that their goodness of fit diverged at extremes. This discrepancy may explain why, as predicted mortality increased, the divergence increased in the Bland-Altman plots. Consequently, these findings may have important implications for understanding variations between ICUs. The Bland-Altman plot demonstrates a substantial degree of difference between the two methods and that, despite their weak linear relationship; the two models are not interchangeable. This result aligns with previous studies that compared models of severity of illness, including Sequential Organ Failure Assessment and Mortality Prediction Model [6], [16].

This disagreement has important implications at individual patient, ICU, and institutional levels, as these models are used to benchmark performance between units and against institutional peers. Moreover, as patients have increasing access to this information, consumers may be influenced by information that is more rightfully placed in the realm of research (to describe populations) and institutional quality (describing trends). Furthermore, given that critically ill patients are at the extremes of predicted mortality, it is important to note that it was these patients who had the most disparate results. Our results do not imply that one model is superior to another, but suggest that, in critically ill patients, administrative models may predict a lower risk of mortality that does not always reflect the individual patient's condition. It is also important to note that how a model assigns mortality may influence provider metrics. For example, in a closed ICU system, critical care physicians may be penalized because the majority of discharges, attributed to them, are deaths, so the best Mortality Index they can legitimately achieve is 1.0. Therefore, as a quality measure, mortality index should be interpreted in this light. In addition, it should be noted that at least in this study of critically ill patients, the mortality index was not normally distributed. Therefore, while it is convenient to report the mean without confidence interval, range, or other statistical description like a box-and-whisker plot, this value provides only limited information even within robust models.

The use of administrative variables for mortality and length of stay prediction is inherently retrospective and reflects the care from admission to discharge, as well as discharge options available. As recently shown by Kozower et al, this approach can skew the predictive model [18]. Iezzoni et al, demonstrated that this methodology creates difficulty in distinguishing if patient outcomes are related to care, severity of illness, or co-morbidities [19]. We attempted to study this concept in our subgroup, which showed improved linear relationship, but continued to show a higher predicted mortality for the physiologic model compared to the administrative model. In our study, the administrative model significantly underestimated the physiologic model predicted mortality for our patient population.

Mortality prediction models are designed to discriminate between survivors and non-survivors at a population level. Predictive modeling is a useful descriptive tool, thereby allowing clinicians to apply the results of trials to their patients and to compare populations between trials. Although these models were developed as research tools, their application has been extended to quality in the form of observed versus expected mortality indices. Subsequently, adjusted mortality indices are intended to allow hospitals to compare themselves to their peers as well as to study temporal differences within the institution. Adjusted mortality has important limitations as a measure of hospital quality, including the “case-mix adjustment fallacy” [20]. “Case-mix adjustment fallacy” is important because severity of illness adjustment is thought to standardize comparisons. Given that only a small proportion of hospital deaths are preventable, the sensitivity of this measure may be decreased when used to estimate quality of an institution or individual unit within an institution [20], [21]. In our sample, the number of “unexpected deaths” as we defined them accounted for a significant portion of deaths (19.8%) in the administrative model, but a smaller proportion (6.9%) using the medical record model. These results suggest that, while using a threshold of 10% predicted mortality, the vast majority of ICU deaths in this sample were predictable. It also suggests that it may be possible to identify these patients. As quality measures improve, standardized mortality ratios may be replaced by more sensitive measures of quality. One possibility would be to move from a mortality index reporting system, to a system of reporting number of unexpected deaths per 1000 patient admissions, which may be more informative. However, at this time, understanding that models differ in how they “standardize risk” is important for policy makers and clinicians.

In this study, we found that well designed models to predict hospital mortality have important limitations that do not diminish their usefulness in describing populations and guiding institutions towards improved quality. However, these models are like apples and oranges - both are good, but unique, and understanding their unique characteristics is critical in the interpretation of their results. Administrative models may have important limitations for critically ill patients, but physiologic models' reliance on clinical variables may mask quality metrics related to “never” events or misadventures in care. Therefore, differences in model performance have important implications as hospitals are increasingly compared to each other, but less so when a hospital compares its own performance over different time points to assess its interventions on quality performance and improvement.

In conclusion, while it has been suggested that administrative models provide estimates of mortality that are similar to physiologic models in non-critically ill patients with pneumonia [12], our results suggest this finding can not be applied to patients admitted to intensive care units. As patients and providers increasingly use publicly reported information in making health care decisions and referrals, it is critical that the provided information be understood. We suggest that when interpreting “report cards” or metrics, health care providers determine how the risk adjustment was made and compares to other risk adjustment models. Furthermore, knowledge of the peer group they are being compared with as well as distribution of the risk adjusted outcome is important in interpreting their own results. We do not suggest that they dismiss the information out of hand as not reflective, but rather use a deeper understanding of their own outcomes to drive improvements in performance that reflect practice improvement.
